# Detection of *bla*_KPC_ gene among carbapenemase producing *Klebsiella pneumoniae* isolated from different clinical specimens at tertiary care hospital of Nepal

**DOI:** 10.1186/s12866-024-03301-9

**Published:** 2024-04-25

**Authors:** Rakshya Baral, Reshma Tuladhar, Sarita Manandhar, Anjana Singh, Samendra Sherchan

**Affiliations:** 1 Central Department of Microbiology, Tribhuvan University, Kathmandu, Nepal; 2 Department of Microbiology, Tri-Chandra Multiple Campus, Tribhuvan University, Kathmandu, Nepal; 3 Department of Biology, Morgan State University, Baltimore, USA; 4 Department of Environmental Health Sciences, Tulane University, New Orleans, USA

**Keywords:** *Klebsiella pneumoniae*, Antibiotic resistant, Carbapenemase, *bla*_KPC_

## Abstract

**Background:**

*Klebsiella pneumoniae* infections have become a major cause of hospital acquired infection worldwide with the increased rate of acquisition of resistance to antibiotics. Carbapenem resistance mainly among Gram negative is an ongoing problem which causes serious outbreaks dramatically limiting treatment options. This prospective cross-sectional study was designed to detect *bla*_KPC_ gene from carbapenem resistant *K. pneumoniae.*

**Materials and Methods:**

A totally of 1118 different clinical specimens were screened and confirmed for KPC producing *K. pneumoniae* phenotypically using Meropenem (10 μg) disc. The *bla*_KPC_ gene was amplified from the isolates of *K. pneumoniae* to detect the presence of this gene.

**Result:**

Of the total samples processed, 18.6% (*n* = 36) were *K. pneumoniae* and among 36 *K. pneumoniae,* 61.1% (n = 22/36) were meropenem resistant. This study demonstrated the higher level of MDR 91.7% (n = 33) and KPC production 47.2% (n = 17) among *K. pneumoniae* isolates. The *bla*_KPC_ gene was detected in 8.3% (n = 3) of meropenem resistant isolates.

**Conclusion:**

Since the study demonstrates the higher level of MDR and KPC producing *K. pneumoniae* isolates that has challenged the use of antimicrobial agents, continuous microbiology, and molecular surveillance to assist early detection and minimize the further dissemination of *bla*_KPC_ should be initiated. We anticipate that the findings of this study will be useful in understanding the prevalence of KPC-producing *K. pneumoniae* in Nepal.

## Background

*Klebsiella pneumoniae* carbapenemase (KPC) is the β-lactamases enzyme of the Ambler class A encoded in *bla*_KPC_ gene predominantly found in *Klebsiella pneumoniae* and other genera of Enterobacteriaceae family [1, 2]. The first KPC producing *K. pneumoniae* was reported in 2001 from a hospital in North Carolina and subsequently witnessed its rise [3, 4]. The KPC encoded gene is present in the transferrable plasmid and synthesize enzyme that hydrolyze wide range of β-lactam antibiotics including penicillin, monobactams, cephalosporin and carbapenems [5]. Different variants of the *bla*_KPC_ gene (KPC-2 to KPC-17) have been detected occasionally in various taxa, such as non-fermenting bacteria. KPC-2 and KPC-3 are the most widely reported and studied variants among KPC-2 to KPC-17 *bla*_KPC_ [6, 7].

The circulation of *bla*_KPC_ gene harboring bacteria in clinical setting is of concern since it is likely to incite imipenem and meropenem resistant strains of bacteria augmenting global antibiotic resistance problem [8]. Among the current risk identified with the spread of carbapenemase enzyme, KPC seems to be pertinent on the ground of misuse and maltreatment of carbapenem antibiotics. This increase in the strains harboring KPC gene can lead to increment in the KPC and OXA-48 strains [9]. At the point when a KPC producer variant becomes a multidrug resistance (MDR), treatment failure of infection due to this strain is likely to increase mortality rates. MDR is a specific type of antimicrobial resistance where a microorganism becomes resistant to at least one antibiotic in three or more antimicrobial categories [10].

The type of antibiotic resistance due to KPC is fast spreading, especially when it is transmitted by transferable carbapenemase-encoding genes, resulting in significant epidemics and severely limiting treatment options. Carbapenemase genes, which can be shared between humans, are most involved. Inadequate empirical antibiotic therapy for severe KPC-KP infections has been linked to higher morbidity and mortality [11]. In Nepal, many studies on phenotypic detection of KPC producing isolates has been carried out, but genotypic studies are minimal. As a result, there is an urgent need to investigate the incidence of major types of genes that cause KPC to spread widely and that clarify the presence of *bla*_KPC_ producing *K. pneumoniae*, in both phenotypically positive and negative isolates. This study is carried out to detect the *bla*_KPC_ gene among the *K. pneumoniae* isolated from different clinical samples at a tertiary care hospital in Kathmandu, Nepal. Production of carbapenemases (KPC) has been the global cause of Carbapenem (Meropenem and Imipenem) resistance among *K. pneumoniae* which is a great therapeutic challenge. The increasing and rapid spread of *bla*_KPC_ gene has not been yet accessed fully in Nepal, so this study was undertaken to ascertain the present scenario of *bla*_KPC_ in gene *K. pneumoniae* isolates obtained through clinical samples in a tertiary hospital in Kathmandu Nepal.

## Materials And Methods

### Study design and bacterial isolation

A prospective study was conducted at Shahid Gangalal National Heart Center (SGNHC) and Central Department of Microbiology, Kathmandu, Nepal from Nov 2018 to Jul 2019. Ethical approval was obtained from the institution of Science and Technology (IOST) by Institutional Review Committee (IRC). A total of 1118 non-duplicate clinical specimens received at Microbiology laboratory of SGNHC were processed. The types of samples collected were urine, pus, sputum, blood, and ET secretion.

*K. pneumoniae* were identified using standard microbiological techniques which included growth on MacConkey Agar, Gram staining and various biochemical tests [12].

### Antimicrobial Susceptibility Test (AST)

Antibiotic susceptibility pattern of isolates was assessed by Modified Kirby-Bauer disk diffusion test on Mueller Hinton agar (MHA) using antibiotics discs; Ampicillin (10 μg), Nitrofurantoin (300 μg), Ciprofloxacin (5 μg), Cotrimoxazole (25 μg), Amoxicillin/Clavulanate (20/10 μg), Ampicillin/Sulbactam (10/10 μg), Cefexime (5 μg), Ceftriaxone (30 μg), Ceftazidime (30 μg), Cefepime (5 μg), Cefotaxime (30 μg), Amikacin (30 μg), Gentamicin (10 μg), Piperacillin/Tazobactam (100/10 μg), Imipenem (10 μg), Meropenem (10 μg), Polymyxin B (10 μg) [13].. Isolates resistant to meropenem disc (zone of dimeter >5 mm) were considered carbapenemase positive and organisms were selected for further testing by using phenyl boronic acid (PBA) for the phenotypic detection of KPC producers and *bla*_KPC_ gene detection [14].

### Phenotypic confirmatory test for KPC producers

The *K. pneumoniae* isolates resistant to Meropenem were subjected combined disc test using carbapenem with and without phenyl boronic acid (PBA) for the phenotypic detection of KPC producers [14]. Briefly, 0.5 McFarland standard suspension of isolate was spread on MHA plates. Meropenem disc was loaded with 20 μl of 20 mg/ml PBA prepared in dimethyl sulphoxide (DMSO) and allowed to dry. Two Meropenem discs, one with PBA and other without PBA was placed on the MHA plates seeded with test organism at the distance of 30 mm and incubated overnight at 37 °C. Isolates with increase of ≥5 mm inhibitory zone around the disc with PBA compared to the disc around Meropenem without PBA was considered KPC producers [15].

### Detection of *bla*_KPC_ gene

The *bla*_KPC_ gene was amplified from the plasmid DNA extracted from all the 36 isolates of *K. pneumonia* by Alkaline hydrolysis method [16]. PCR amplification was performed using the primers for *bla*_KPC_ i.e., forward primer: 5’ACGACGGCATAGTCATTTGC 3′ and reverse primer: 5′ CATTCAAGGGCTTTCTTGCTGC 3′ with amplicon of 538 base pairs [17]. PCR mixture of total volume 25 μl was prepared which consisted of 3 μl of template DNA, 0.5 μl each of forward and reverse primer and 21 μl of PCR master mix. The thermal cycling conditions for amplification was initial denaturation at 95 °C for 15 minutes followed by 32 cycles of denaturation at 94 °C for 30 seconds, annealing at 58 °C for 90 seconds and extension at 72 °C, followed by final extension at 72 °C for 10 minutes. The amplified PCR products were analyzed in 1.2% agarose gel stained with ethidium bromide.

### Data analysis

All the data were entered and analyzed statistically using statistical package for social science (SPSS) software (version 25). Binary variables were compared using chi-square test. A probability (p) value of <0.05 was considered significant.

## Results

### Distribution of Carbapenemase producing *K. pneumoniae* with respect to different variables

Among 1118 different clinical specimens investigated, growth was observed in 17.2% (*n* = 193) specimens where 18.7% (*n* = 36) isolates were identified as *K. pneumoniae.*

Distribution of *K. pneumoniae* in Clinical Specimens.

**Table:** 

Total Specimens	Specimens with growth	Growth percentage	*K. pneumoniae* isolates	Percentage of *K. pneumoniae* isolates
1118	193	17.2	36	18.65%

Out of 36 *K. pneumoniae*, 47.2% (*n* = 17) were isolated from urine sample followed by 22.2% (n = 17) from sputum sample. Similarly, the highest numbers of isolate were recovered from inpatient (Table 1).

**Table 1:** Distribution of Carbapenemase producing *K. pneumoniae* (with respect to different variables)

Variables	*K. pneumoniae* (N, %)	KPC *K. pneumoniae* (N, %)
Patient status		
Inpatients	19 (52.8)	14 (82.4)
Outpatients	17 (47.2)	3 (17.6)
Total	36	17
Sample type		
Urine	17 (47.2)	9 (53.0)
Pus	7 (19.4)	3 (17.6)
Sputum	8 (22.2)	4 (23.5)
Blood	2 (5.6)	1 (5.9)
ET Secretion	2 (5.6)	0 (0)
Total	36	17

### Antibiotic susceptibility profile of *K. pneumoniae*

Antibiotic susceptibility profile showed that 91.7% (n = 33) and 86.1% (n = 31) of total *K. pneumoniae* were resistant to ciprofloxacin and cephalosporins respectively. Likewise, 61.1% (n = 22) and 69.4% (n = 25) *K. pneumoniae* were resistant to Meropenem and Imipenem, respectively and 91.7% (n = 33) were multi-drug resistance (Table 2).

**Table 2:** Antibiotic susceptibility pattern of *K. pneumonia* (n = 36)

Antibiotics	Class	Potency	Susceptible N (%)	Resistant N (%)
Nitrofurantoin	Imidazolidinedione	300 μg	5(13.9)	12(33.3)
Ciprofloxacin	Fluoroquinolone	5 μg	3(8.3)	33(91.7)
Cotrimoxazole	Sulphonamide	25 μg	18 (50)	18 (50)
Amoxicillin/ Clavulanic Acid	β-lactam/ β-lactamase inhibitor combination	20/10 μg	11(30.6)	25(69.4)
Ampicillin/Sulbactum	β-lactam/ β-lactamase inhibitor combination	10/10 μg	11(30.6)	25(69.4)
Cefixime	Third generation Cephalosporin	5 μg	9(25)	27(75)
Ceftriaxone	Third generation Cephalosporin	30 μg	9(25)	27(75)
Ceftazidime	Third generation Cephalosporin	30 μg	9(25)	27(75)
Cefepime	Fourth generation Cephalosporin	5 μg	8(22.2)	28(77.8)
Cefotaxime	Third generation Cephalosporin	30 μg	7(19.4)	29(80.6)
Amikacin	Aminoglycoside	30 μg	15(41.7)	21(58.3)
Gentamicin	Aminoglycoside	10 μg	12(33.3)	24 (66.7)
Piperacillin/ Tazobactum	β-lactam/ β-lactamase inhibitor Combination	100/10 μg	12(33.3)	24(66.7)
Imipenem	Carbapenem	10 μg	11 (30.6)	25(69.4)
Meropenem	Carbapenem	10 μg	14(38.9)	22(61.1)

### Comparison of Meropenem susceptibility with KPC production

Among 14 Meropenem sensitive *K. pneumoniae* isolates, none of them were KPC producers whereas 17 *K. pneumoniae* isolates among 22 Meropenem resistant *K. pneumoniae* isolates were found to be KPC producers (Table 3).

**Table 3:** KPC producers among Meropenem resistant *K. pneumoniae*

Tested Antibiotic	Confirmation	KPC Production		Total (N, %)
		Positive (N, %)	Negative (N, %)	
Meropenem	Sensitive	0 (0)	14 (73.7)	14 (38.9)
	Resistant	17 (100)	5 (26.3)	22 (61.1)
Total		17 (47.2)	19 (52.8)	36 (100)

### Meropenem resistant *K. pneumoniae* with *bla*_KPC_ gene

Among 22 Meropenem resistant K*. pneumoniae* isolates, two harbored *bla*_KPC_ gene and one Meropenem sensitive isolate also harbored *bla*_KPC_ gene (Table 4).

**Table 4:** Meropenem resistant *K. pneumoniae* harboring *bla*_KPC_ gene

*bla*_KPC_ Detection	Meropenem		Total (%)
	Sensitive (N, %)	Resistant (N, %)	
Positive	1(7.1%)	2(9.1%)	3(8.3%)
Negative	13(92.9%)	20(90.9%)	33(91.7%)
Total	14(38.9%)	22(61.1%)	36(100%)

### Association of KPC producer and the presence of *bla*_KPC_ gene

Among the 17 KPC positive isolates, only 11.8% (n = 2) of them harboured *bla*_KPC_ gene while rest were negative for *bla*_KPC_ gene. No significant association was found between carbapenemase production and presence of *bla*_KPC_ gene (P > 0.05) (Table 5).

**Table 5:** Comparison of KPC positive with *bla*_KPC_ gene detection

Carbapenemase Production	*bla*_KPC_ detection		Total (%)	P-value
	Positive (N, %)	Negative (N, %)		
Positive	2 (66.67)	15 (45.45)	17 (47.22)	0.481*
Negative	1 (33.3)	18 (54.55)	19 (52.78)	
Total	3 (8.33)	33 (91.67)	36 (100)	

### PCR amplification of *bla*_KPC_ gene in Meropenem resistant isolates

**Fig. 1:**
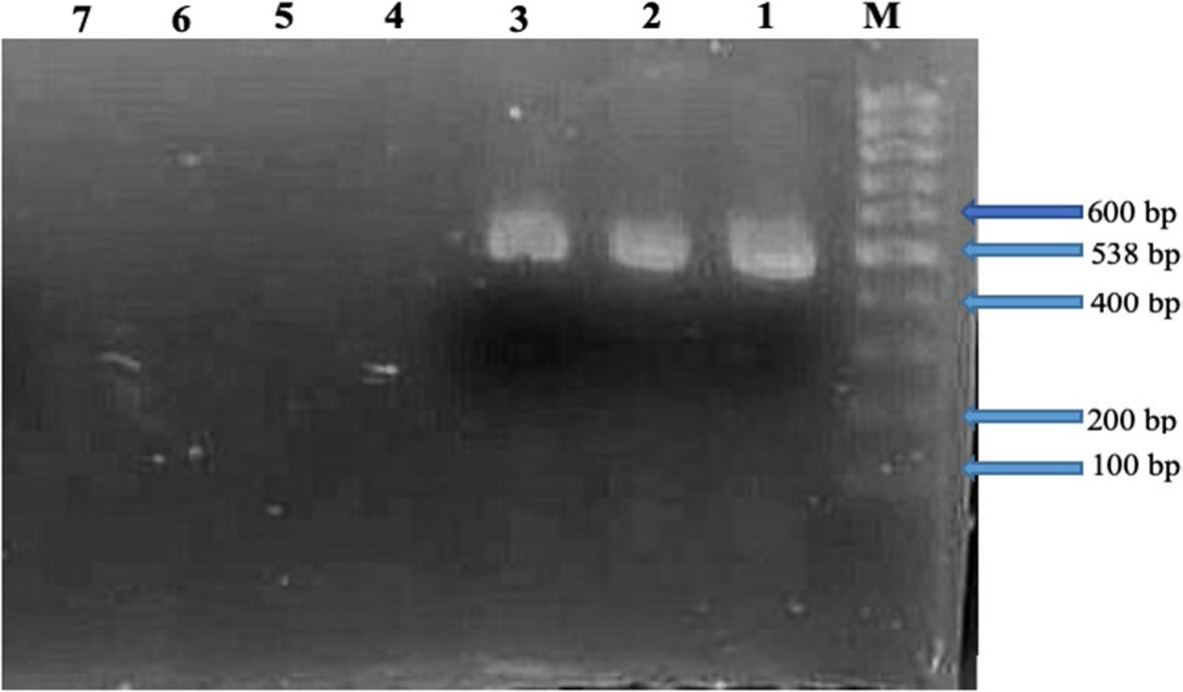
This study evaluated the isolated samples by conducting agarose gel electrophoresis on the PCR products amplified using primer designed for *bla*_KPC_ gene. Lane M, 1 kb DNA size marker (100-1000 bp); Lane 1/2/3: *bla*_KPC_ positive isolates, Lane 4/5/6/7: *bla*_KPC_ negative isolates

The primers designed to target the *bla*_KPC_ gene successfully amplified a 538 base pairs fragment in 3 out of the 36 tested isolates using polymerase chain reaction. Fig. 1 illustrates the outcomes of the amplification process.

## Discussion

Low culture positivity of *K. pneumoniae* has been reported in this study which agrees with other studies [11, 18, 19]. The use of antibiotics prior to sample collection, control of bacterial infection within the hospitals as well as success of infection control strategy are the common factors behind the low growth rate observed [20]. The total number of growth positive isolates of *K. pneumoniae* in this study was comparable with the study done by Nepal et al. [18].

This research aimed to detect the *bla*_KPC_ gene in SGNHC, as individuals with heart conditions are at a heightened risk of developing severe infections that can have a significant impact on their healing and overall health. This hospital also has huge patient flow patients including a diverse range of cases, which makes it a suitable sample for studying bacterial infections in Nepal. Therefore, the research intends to enhance infection control procedures, which could result in better patient results, shorter hospital stays, and decreased healthcare expenses.

Similar to Lidd et al. [21], greater number of *K. pneumoniae* isolates were received from inpatient department in this study. High number of isolates from inpatient departments is because these *K. pneumoniae* are often associated with hospital acquired infections [21]. Similarly, higher percentage of KPC was found in inpatient department which is in concordance to other studies [22] [23]. The Enterobacteriaceae family, particularly *K. pneumoniae*, is one of the most common causes of nosocomial infections.

At the time of admission to the inpatient department like ICU, a longer stay in hospital is a risk factor for KPC colonization. The use of carbapenems at ICU admission (starting 48 hours before ICU admission), upper digestive endoscopy, and transfer from another hospital are also the risk factors.

In this study, *K. pneumoniae* was found to be isolated in higher number from urine followed by sputum samples. This was in accordance with other study in Nepal [11]. Various studies show that *K. pneumoniae* is predominantly isolated from urine sample [24–26]. *K. pneumoniae* being one of the common causative agents of UTI, its isolation is very common from the urine sample [26]. However, Biradar and Roopa [27] reported the highest percentage of *K. pneumoniae* from pus followed by urine.

High percentage of *K. penumoniae* isolates were resistant to third-generation cephalosporin antibiotics i.e., Cefixime, Cefotaxime, Ceftazidime, Ceftriaxone, and fourth generation cephalosporin Cefepime. Increased resistance to third generation cephalosporin antibiotics has been described by previous studies also [28–30]. A study conducted by Adhikari et al. [11] and Lohani et al. [31] also reported more than 90.0% of the isolates being resistant to these antibiotics. The widespread use of cephalosporin antibiotics without knowing the severity of infection could explain the increased resistance towards these antibiotics [32].

In addition, reduced susceptibility towards carbapenem was observed in this study like that reported by Estabraghi et al. [18, 33–35]. Meropenem and Imipenem had the highest resistance levels. Shrestha et al. [17] found a higher rate of Meropenem and Imipenem resistance in *K. pneumoniae*. The presence of an analogously significant fraction of carbapenem-resistant clinical isolates is indicated by these resistance patterns. Because there has been very little research done in Nepal to discover carbapenemase producers, it is impossible to assess whether the trend of carbapenemase producers is increasing or decreasing. The increasing trend of using cephalosporin and carbapenem directly for treatment of infection caused by MDR isolates is major problem in developing countries like Nepal [36]. Multiple mechanisms developed by organism are responsible for rise in resistance towards carbapenem such as production of β-lactamases, blocking the entry of these antibiotics, or efflux pumps [37–39]. However, polymyxin B has been found to be the choice of treatment which is considered effective to treat the infections caused by MDR Gram-negative bacteria in adult patients [40]. Factors like poor regulation of antibiotics without prescription, self-medication and lack of laboratory facilities contribute to increasing rates of antibiotic resistance [41, 42]. The resistance among pathogens is also mediated by horizontal spread of clones of resistant bacteria, within hospitals and nursing homes, as well as facilitated to some extent by migration and international mobility [43].

High number of *K. pneumoniae* were found to be MDR. Variation in the range of MDR *K. pneumonia* was detected extending from 3.0–100% from studies in Nepal [11, 18, 28, 30, 44–46]. Poor hygiene, misuse of antibiotics and absence of antimicrobial surveillance program are the common risk factors associated with the development of MDR [47, 48]. The rising incidence of the clinical MDR-KPC phenotype has been linked to greater mortality rates, constituting a significant public health issue [49]. Hence, carbapenem resistance has become a serious global problem. These drugs are generally used as last resort for serious Gram-Negative infections like infections caused by *K. pneumoniae* [50, 51].

The phenotypic method was used to screen KPC producers among carbapenem resistant isolates. Nearly half of the isolates were detected as KPC producers which is like the result obtained by Shrestha et al. [17]. Boronic acid tests using Imipenem, or Meropenem as an antibiotic substrate demonstrated an excellent ability to differentiate KPC enzymes [52]. The elevated frequency of KPC positive isolates in present study might be due to the increased use of carbapenem.

Among total Meropenem sensitive *K. pneumoniae* isolates, none of them were KPC producers whereas almost half of *K. pneumoniae* isolates were found to be KPC producers among Meropenem resistant *K. pneumoniae* isolates. This result is higher than that reported by Foschi et al. [53] (56.36%). It’s possible that high frequencies of several types of carbapenemase producers exist in some places, such as Greece (KPC and VIM) and the Indian subcontinent (KPC, NDM, OXA-181) [54]. *K. pneumoniae* plays a vital role since it has been continuously detected as most prevalent species of Enterobacteriaceae for propagating ESBL genes in hospitals over past 30 years. It could also have a role in the spread of carbapenemase production in patients with similar risk characteristics (patients who are receiving broad-spectrum antibiotherapy, immunocompromised patients, patients in intensive care units, transplant patients, and surgical patients) [55].

In the study, all the isolates of *K. pneumoniae* were subjected to molecular characterization for *bla*_KPC_ and the total of 8.3% (*n* = 3) harbored the corresponding gene. Shrestha et al. [17]reported 6.45% of *bla*_KPC_ carbapenemase resistant gene among Meropenem resistant isolates. However, Bina et al. [9] showed 80.5% isolates were positive phenotypically and all of them possessed *bla*_KPC_ genes. Only 2 Meropenem resistant isolates have 2 *bla*_KPC_ gene. This suggests that a carbapenemase other than KPC is present in other isolates. One isolate from Meropenem sensitive isolate harbored *bla*_KPC_ gene. The acquisition of carbapenemase-encoding genes is not always linked to high levels of carbapenem resistance [56]. Several variables can account for this varied susceptibility, as the presence of additional resistance mechanisms [57]; genetic suppression resulting in a silenced gene; plasmid copy number-dependent gene dosage [58]Therefore, it is reported that carbapenemase gene is detected with higher sensitivity by molecular approach [58].

One *K. pneumoniae* isolate that was phenotypically sensitive to Meropenem harbored *bla*_KPC_ gene. Shrestha et al. [17]have reported that there might be chance of expression of these genes in isolates but not phenotypically expressed. Also, it might not be expressed in phenotypically resistant isolates in some cases. Shrestha et al. [17]. Likewise, Kitchel et al. [58] have mentioned that the number of copies of *bla*_KPC_ in the upstream genetic environment, as well as deletions in the upstream genetic environment, may impact the level of KPC production. Similarly, another relevant finding showed that no resistance was seen to carbapenems by isolate which were positive for *bla*_KPC_ gene. Similar results were reported by Peleg et al. [59] that showed only 5 out of 19 isolates that carried carbapenemases genes expressed resistance to carbapenems.

Among 17 KPC producers, only two isolates harbored *bla*_KPC_ gene. Even though 17 *K. pneumoniae* isolates tested positive for the KPC gene using PBA in this study, only three of them had the *bla*_KPC_ gene. Because boronic acid derivatives are effective inhibitors of these enzymes, the false-positive results could be attributed to the synthesis of AmpC beta-lactamases or some CTXM beta-lactamases [60]. As a result, noting the false-positive result during phenotypic confirmation is extremely important. Further negative results from molecular analysis do not necessarily imply that those isolates lack the genes of our choice; they may have those genes but are unable to express them [17].

Antibiotic resistance is increasing, according to the studies, and medical societies are rapidly running out of treatment alternatives. This is causing a significant issue in the pharmacopeia. Early detection of these sorts of resistance genes, such as KPC would be a beneficial tool for identifying infections and aiding in their control and prevention. As a result, it is critical for all laboratories to become aware of such infections, which may pose a hazard to public health if not treated and controlled promptly [17]. Furthermore, the clonal expansion of *bla*_KPC_ gene reported in various epidemics suggests that infection management for this organism is challenging. Worse, because of their antibiotic resistance, treating infections caused by *K. pneumoniae* is extremely challenging, resulting in high fatality rates. KPCs are found in several Gram-negative bacteria, not just *Klebsiella* spp. It is important to detect KPC gene to improve the quality of health care, to minimize the prevalence of infections and the emergence of carbapenem resistance in these bacteria and other Gram-negative pathogens. Consequently, phenotypic, and genotypic identification techniques should be used for precise diagnosis and study even if KPC positive *K. pneumoniae* is not significantly linked to *bla*_KPC_ gene.

The *bla*_KPC_ genes that code for the KPC enzymes are frequently flanked by transposon-related sequences found on transferable plasmids, giving them the ability to spread quickly. Because of its position on plasmids, the KPC family has the greatest potential for spread, especially as it is most frequently seen in *K. pneumoniae*, an organism known for its propensity to acquire and transfer resistance determinants. Although KPC-lactamases are most detected in *K. pneumoniae*, these enzymes have also been found in *Enterobacter* spp. and *Salmonella* spp. [2]. Hence, the presence of gene *bla*_KPC_ encoding KPC enzyme can later result in production of KPC enzyme.

### Strengths and limitations

This research is the first comprehensive investigation to determine the occurrence of *bla*_KPC_ gene in *K. pneumoniae* using both phenotypic and genetic techniques. The result of this study can be valuable to many major tertiary hospitals with high rates of hospital-acquired infections. These results can help shape the antimicrobial guidelines for tertiary care facilities in developing strategies for managing hospital infections, deciding on treatment plans, and determining diagnostic procedures. Limitation of this research includes the short duration of the study, a smaller number of participants, and its focus on a single medical facility. A longitudinal study can be carried out at multiple tertiary care centers for future research. This study is going to be an invaluable resource for future research on *bla*_KPC_ in Nepal, as this study can become an important reference for future studies on *bla*_KPC_ in Nepal.

## Conclusion

This study demonstrates the higher level of MDR and KPC producing *K. pneumonia* isolates that has challenged the use of antimicrobial agents. Molecular approaches aimed at detecting strains harboring carbapenemase genes are highly sensitive and efficient for confirmation of cases. This study also showed that phenotypically Meropenem sensitive isolates does not mean that they are KPC non-producers. Conversely, there are no proper phenotypic methodologies to routinely conduct clinical and laboratory finding of KPC producers. In this respect, the presence of the *bla*_KPC_ gene must be confirmed by molecular biology techniques to define the production of *bla*_KPC_ gene. To prevent emergence and spread of *bla*_KPC_ gene, an antimicrobial policy must be developed and strictly enforced, as well as proper infection control measures.

Findings of this study will be useful in developing and implementing an efficient infection disease control strategy in Nepal to avoid and reduce the prevalence of KPC-producing *K. pneumoniae.* Carbapenem resistance in *K. pneumoniae* has been detected in clinical settings, hence continuous microbiology, and molecular surveillance to assist early detection and minimize the further dissemination of *bla*_KPC_ should be initiated.

## Data Availability

The datasets used and/or analyzed during the current study are available from the corresponding author on reasonable request.

## Abbreviations

KPC: *Klebsiella pneumoniae* carbapenemase
SGNHC: Shahid Gangalal National Heart Center
IOST: Institution of Science and Technology
IRC: Institutional Review Committee
MHA: Mueller Hinton agar
PBA: Phenyl Boronic Acid
DMSO: dimethyl sulphoxide
MDR: Multidrug resistance
ICU: Intensive Care Unit
VIM: Verona integron-encoded metallo β-lactamase
NDM: New Delhi metallo beta lactamase 1
ET Secretion: Endotracheal Secretion
